# Prediction modelling of inpatient neonatal mortality in high-mortality settings

**DOI:** 10.1136/archdischild-2020-319217

**Published:** 2020-10-22

**Authors:** Jalemba Aluvaala, Gary Collins, Beth Maina, Catherine Mutinda, Mary Waiyego, James Alexander Berkley, Mike English

**Affiliations:** 1 Health Services Unit, KEMRI-Wellcome Trust Research Programme, Nairobi, Kenya; 2 Paediatrics and Child Health, University of Nairobi, Nairobi, Kenya; 3 Centre for Tropical Medicine and Global Health, Nuffield Department of Medicine, University of Oxford, Oxford, UK; 4 Centre for Statistics in Medicine, Nuffield Department of Orthopaedics, Rheumatology and Musculoskeletal Sciences, Botnar Research Centre, University of Oxford, Oxford, UK; 5 Oxford University Hospitals, NHS Foundation Trust, Oxford, UK; 6 Pumwani Maternity Hospital, Nairobi, Kenya; 7 KEMRI-Wellcome Trust Research Programme, Kilifi, Kenya; 8 The Childhood Acute Illness & Nutrition (CHAIN) Network, P.O Box 43640 – 00100, Nairobi, Kenya

**Keywords:** neonatology, mortality

## Abstract

**Objective:**

Prognostic models aid clinical decision making and evaluation of hospital performance. Existing neonatal prognostic models typically use physiological measures that are often not available, such as pulse oximetry values, in routine practice in low-resource settings. We aimed to develop and validate two novel models to predict all cause in-hospital mortality following neonatal unit admission in a low-resource, high-mortality setting.

**Study design and setting:**

We used basic, routine clinical data recorded by duty clinicians at the time of admission to derive (n=5427) and validate (n=1627) two novel models to predict in-hospital mortality. The Neonatal Essential Treatment Score (NETS) included treatments prescribed at the time of admission while the Score for Essential Neonatal Symptoms and Signs (SENSS) used basic clinical signs. Logistic regression was used, and performance was evaluated using discrimination and calibration.

**Results:**

At derivation, c-statistic (discrimination) for NETS was 0.92 (95% CI 0.90 to 0.93) and that for SENSS was 0.91 (95% CI 0.89 to 0.93). At external (temporal) validation, NETS had a c-statistic of 0.89 (95% CI 0.86 to 0.92) and SENSS 0.89 (95% CI 0.84 to 0.93). The calibration intercept for NETS was −0.72 (95% CI −0.96 to −0.49) and that for SENSS was −0.33 (95% CI −0.56 to −0.11).

**Conclusion:**

Using routine neonatal data in a low-resource setting, we found that it is possible to predict in-hospital mortality using either treatments or signs and symptoms. Further validation of these models may support their use in treatment decisions and for case-mix adjustment to help understand performance variation across hospitals.

**What is already known on this topic?**

- Existing neonatal prognostic models are suited for advanced care settings as they use parameters that are not available in routine practice in low-resource settings.
- These parameters include pulse oximetry, blood gases and other laboratory tests.

**What this study adds?**

- Application of recent methodological guidance; Prognosis Research Strategy and Transparent reporting of a multivariable prediction model for individual prognosis or diagnosis.
- Using routine neonatal data in a low-resource setting, we found that it is possible to accurately predict in-hospital mortality.
- Further validation of these models may support their use in treatment decisions and for case-mix adjustment to help understand and improve performance variation across hospitals.

## Introduction

Kenya and other low-income to middle-income countries (LMICs) accounted for 99% of global neonatal mortality in 2017. Improved delivery of essential interventions in hospitals is expected to play a key role in achieving Sustainable Development Goal 3.2—a neonatal mortality rate of 12 per 1000 live births or lower.^1–3^ A better understanding of hospitals’ neonatal mortality and reliable and timely information on how this varies may support efforts to improve hospital care at scale.^4 5^

Well-performing prognostic models can aid clinical decisions and at health system level may support better decisions to improve services.^6^ We reviewed existing neonatal prognostic models, and these predominantly use physiological parameters (eg, blood gas values) that are not available in routine practice in LMICs.^7^ However, the use of treatment data is a potentially useful approach to predicting in-hospital neonatal mortality in LMICs, given its greater availability.^7^ Current models that use treatments as predictors typically focus on those given in intensive care, and there are limitations in the methods used to develop and validate them.^7^ These limitations may be addressed by the selection of treatments relevant to the LMIC setting and application of recommended approaches to prognostic model development and validation.^8^

Candidate treatment predictors are essential interventions included in clinical practice guidelines for in-hospital neonatal care developed by the WHO and adapted for Kenya.^9^ Alternative predictors are simple clinical signs recommended for use in routine practice in Kenya,^9^ and we have recently shown data on these candidate predictors can be collected.^10^

Our aim was to use these data to develop and validate two models to predict in-hospital neonatal mortality, the Neonatal Essential Treatment Score (NETS) and the Score of Essential Neonatal Symptoms and Signs (SENSS). These data could be combined in one model but as head-to-head comparisons of prognostic models in the same population are rare, we aimed as a start to evaluate which approach might be most appropriate (based on data availability and performance) for the population of interest.^11^

## Methods

### Source of data

Data were obtained from a routine data collection system described in detail elsewhere.^12^ At the time of this study, neonatal unit data were from one hospital—a large urban maternity hospital in Nairobi County, Kenya.^12^ The unit provides essential inpatient care to 60 neonates at any given time with approximately 4500 admitted annually.

### Participants

Two data sets were extracted for this study. The first included admissions from April 2014 to December 2015 (n=9115) and the second included admissions from January 2016 to July 2016 (n=2735). The data capture system randomly selected 60% of the cases for collection of a comprehensive set of clinical and treatment data (the full data set), while for 40% of admissions, fewer variables were collected (the minimum data set). The full data sets from the first (n=5427) and second (n=1627) periods were used for model derivation and validation, respectively.

### Outcome

The outcome was all-cause in-hospital neonatal unit mortality. Outcomes should be assessed blind to predictors to avoid influencing outcome assessment.^8^ The data collectors were not blinded but were unaware of these prognostic model analyses.

### Predictors

The treatment predictors included in NETS were use/non-use of supplementary oxygen, enteral feeds, first-line intravenous antibiotics (penicillin and gentamicin) and parenteral phenobarbital (online supplemental table 1).^9^ Continuous positive airway pressure, phototherapy, exchange transfusion and kangaroo mother care were rarely prescribed (<1.5%) at admission and were omitted.

10.1136/archdischild-2020-319217.supp1 Supplementary data

For the SENSS model, presence or absence of difficulty feeding, convulsions, indrawing, central cyanosis and floppy/inability to suck were included (online supplemental table 2).^9^ We excluded temperature and respiratory rate as these were poorly documented (89% and 65% missingness, respectively). We also excluded bulging fontanelle as it was a rare sign (present in 0.2% of cases in the derivation data set).

We also included birth weight by category (<1 kg, 1.0–<1.5 kg, 1.5–<2.5 kg, 2.5–4.0 kg and >4 kg) and sex in both models. Weight as a continuous predictor is preferable to avoid information loss. However, these categories are based on a priori clinical consensus rather than data driven.^8 9^ Gestation at birth was not included due to 70% missingness. This rate of missingness is consistent with previous work in Kenyan hospitals.^13^

### Sample size

Seven predictors (six binary and one categorical) were included in each model. The five birthweight categories required four parameters to be calculated. There was thus a total of 10 parameters in each model against 445 deaths, giving 45 deaths per variable (445/10). This exceeds the recommended ratio of 10 events per prognostic variable and the more recently suggested ratio of 20 events per variable for model derivation.^14^ The external validation data set contained 151 in-hospital deaths and 1476 non-events (no deaths). This is more than the minimum recommended 100 events and 100 non-events for validation studies.^15^

### Missing data

For NETS derivation, 587 out of the 5427 observations were excluded as treatment sheets were missing in the patient files (online supplemental table 3), leaving 4840 with 447 in-hospital deaths (9%). In the validation data set, 143 out of 1627 observations had no treatment sheets (online supplemental table 4), leaving 1443 with 137 in-hospital deaths (9.5%). We considered multiple imputation inappropriate, given that entire treatment sheets were missing which would necessitate imputation based on a limited set of non-treatment data such as clinical symptoms.

Predictor missingness in the SENSS derivation data set (online supplemental table 5) ranged from 0.2% (sex and birth weight) to 16% (floppy/unable to suck). In the validation data set, missingness (online supplemental table 5) ranged from 0.1% (sex) to 14% (severe indrawing). We assumed a missing at random (MAR) mechanism for the observed missingness. Multiple imputation using the chained equation approach was implemented for both data sets.^16^

### Statistical analysis methods

Logistic regression without variable selection was used. We derived SENSS using 31 imputed data sets (based on 31% of observations missing at least one variable). Parameter estimates were then combined using Rubin’s rule.

Model calibration was assessed by plotting the predicted probability of in-hospital death against the observed proportion. Discrimination was assessed by the c-statistic (equivalent to the area under the receiver operating curve).^15 17^ The prediction models were internally validated using bootstrapping to assess any overfitting.

Temporal external validation was done by applying the model coefficients obtained at derivation to the external validation data. For SENSS, this required imputation of 23 data sets (23% of the cohort had one or more missing values).

## Results

The clinical characteristics of all eligible patients are shown in table 1. Slightly more than half of the neonates were male in the derivation and validation data sets. The majority (70%) had a normal birth weight (2.5–4.0 kg), and less than 5% were born outside the facility.

**Table 1:** Characteristics of patients included in model derivation and external validation

Characteristic	Derivation				External validation			
	NETS, n=4840		SENSS, n=5427*		NETS, n=1443		SENSS, n=1627*	
	n†	%	n†	%	n†	%	n†	%
Sex								
Male	2605	54	2942	54	850	59	962	60
Missing	12	0.3			2	0.1		
Birth weight (kg)								
<1	31	0.6	32	0.6	10	**1**	10	**1**
1.0–<1.5	115	2	136	**3**	40	**3**	45	**3**
1.5–<2.5	1043	22	1182	22	316	22	361	22
2.5–4.0	3438	71	3848	71	1002	69	1126	69
>4.0	204	4	229	4	74	5	85	5
Missing	9	0.2			1	0.1		
Mode of delivery								
Spontaneous vaginal	2697	57	3107	57	897	63	1018	63
Assisted vaginal	1	0.02	6	0.1	0	0	1	0.1
Breech	40	1	102	2	19	1	34	2
Caesarean section	1999	42	2212	41	509	36	574	35
Missing	145	3			18	1		
Outborn§‡								
Yes	107	2	123	2	57	4	60	4
Missing	0	**0**			0	**0**		
HIV exposure								
Exposed	287	6	338	6	74	5	93	6
Missing	277	6			80	6		
Outcome								
Alive	4374	90	4918	91	1300	90	1476	91
Dead	447	9	509	9	137	9	151	1
Missing	19	0.4			6	0.4		

*Data presented are after multiple imputation. The multiple imputation filled in the missing values while preserving the pattern of distribution observed in the original data sets (online supplemental table 8).

†Denominators for the variables obtained by subtracting the missing data from the sample (4840 for derivation, 1443 for external validation)

‡Outborn refers to neonates admitted to the unit having been born either in another facility, at home or on the way to hospital

NETS, Neonatal Essential Treatment Score; SENSS, Score for Essential Symptoms and Signs.

### Derivation model specification

A complete case regression analysis was used for the treatment model. A total of 19 patients were missing outcome, 9 were missing birth weights and 12 were missing sex, leading to a total of 40 observations omitted (table 1). The derivation model therefore included 4800 observations with 445 deaths.

### Model results

Box 1 shows the final equations for NETS and SENSS models in the log odds scale.

Box 1 **Logistic regression models for NETS and SENSS**

**NETS**

$$\begin{array}{ll} Linearpredicator= & -4.1521+5.6836*ELBW+VLBW+1.4186*LBW-\\ & 0.2927*Macrosomia-0.3125*Male+1.3695*Antibiotics+\\ & 1.3256*Fluids-1.9135*Feeds+0.6142*Oxygen+\\ & 2.5947*Phenobarbital \end{array}$$

**SENSS**

$$\begin{array}{ll} Linearpredictor= & -3.8583+5.7580*ELBW+3.7082+VLBW+0.9232*LBW-\\ & 0.4918*Macrosomia-0.1336*Male+1.3596*Difficultyfeeding+\\ & 1.3977*Convulsion+1.9790*Indrawing+0.9584*Cyanosis+\\ & 1.6266*Floppyunabletosuck \end{array}$$

For each variable, the presence of the indicator takes a value of 1 and absence takes a value of 0. The coefficients are summated to give the linear predictor, which is then converted to predicted probability of in-hospital mortality.ELBW, extremely low birth weight; LBW, low birth weight; NETS, Neonatal Essential Treatment Score; SENSS, Score of Essential Neonatal Symptoms and Signs; VLBW, very low birth weight.

### Apparent performance

**Figure 1:**
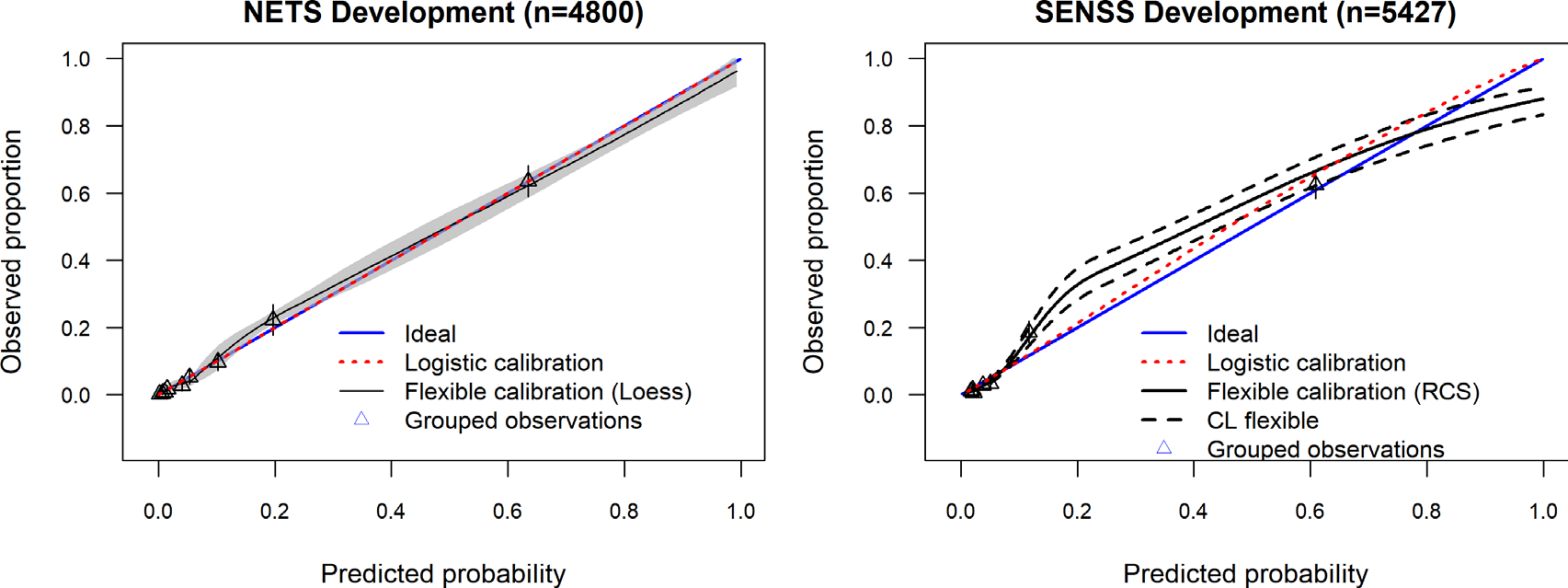
Calibration plot for NETS and SENSS in the derivation data sets. NETS, Neonatal Essential Treatment Score; SENSS, Score for Essential Neonatal Symptoms and Signs;RCS, Restricted Cubic Splines;CL, Confidence Limits (95%).

Figure 1 shows model apparent performance in the derivation data sets. The c-statistic (model discrimination) for NETS was 0.92 (95% CI 0.90 to 0.93) and that for SENSS was 0.91 (95% CI 0.89 to 0.93). The predicted probability of in-hospital death for patients who died was therefore higher than for those who left the unit alive 92% and 91% of the time for the NETS and SENSS, respectively.

### Internal validation

There was a small difference between the apparent performance and the optimism-corrected performance, indicating negligible overfitting (table 2). The original coefficients were therefore taken forward for external validation without shrinkage.

**Table 2:** Evaluation of the NETS and SENSS models for optimism after bootstrapping

Parameter	Calibration				Discrimination*	
	Intercept		Slope			
	NETS	SENSS	NETS	SENSS	NETS	SENSS
Original	0	0	1	1	0.918	0.902
Corrected	−0.062	−0.029	0.979	0.986	0.916	0.901

*c-statistic of the logistic regression model.

NETS, Neonatal Essential Treatment Score; SENSS, Score for Essential Symptoms and Signs.

### External validation

For NETS, there was a higher proportion of prescription of all the five treatments in the external validation data set compared with the derivation data set (online supplemental table 6). A smaller proportion of deaths was observed in those who had a prescription of intravenous antibiotics, intravenous fluids, oxygen and phenobarbital in the external validation data set.

For SENSS, among the patients who died, the proportion with clinical signs present was higher in the derivation data set compared with the external validation data set for four out of the five signs included as predictors (online supplemental table 7).

**Figure 2:**
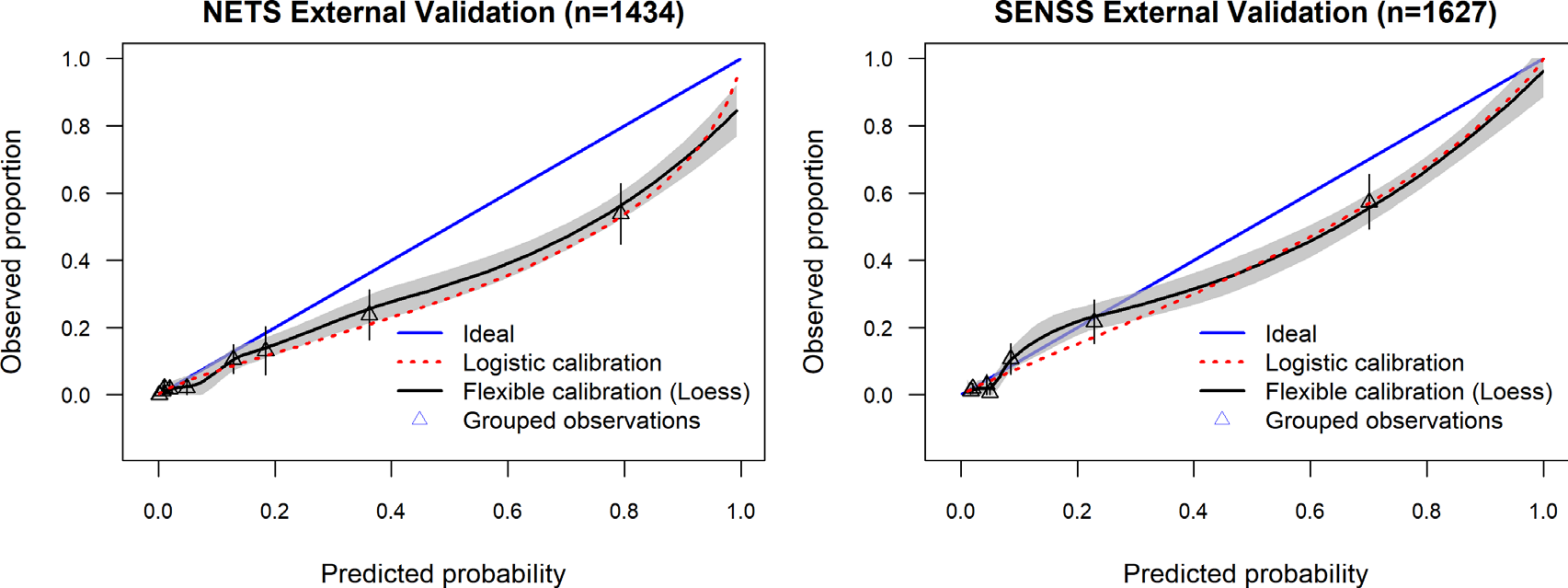
Calibration curves for NETS and SENSS in the external validation data sets. NETS, Neonatal Essential Treatment Score; SENSS, Score for Essential Neonatal Symptoms and Signs.

The two models demonstrated similar discrimination at external validation (figure 2). The c-statistic was 0.89 (95% CI 0.86 to 0.92) for NETS (compared with 0.92 after internal validation) and 0.89 (95% CI 0.84 to 0.93) for SENSS (compared with 0.91 after internal validation). However, greater deterioration was observed for calibration. The calibration intercept dropped to −0.72 (95% CI −0.96 to −0.49) for NETS and −0.33 (95% CI −0.56 to −0.11) for SENSS.

## Discussion

The NETS and SENSS models demonstrated similar performance at derivation; c-statistic (discrimination) for NETS was 0.92 (95% CI 0.90 to 0.93) and that for SENSS was 0.91 (95% CI 0.89 to 0.93). There was minimal overfitting at derivation for both. At external validation, NETS had a c-statistic of 0.89 (95% CI 0.86 to 0.92) and SENSS had a c-statistic of 0.89 (95% CI 0.84 to 0.93), while the calibration intercept for NETS dropped to −0.72 (95% CI −0.96 to −0.49) and that for SENSS dropped to −0.33 (95% CI −0.56 to −0.11).

Model predictors were preselected based on their availability in clinical practice.^9 18^ No selection procedures were employed for two reasons. First, the ideal case is a limited number of preselected predictors entered into a full model.^15^ In addition, a more parsimonious model (fewer predictors) may be expected to have better predictive performance.^15^ However, besides the 0.8 threshold for good discrimination, there is no consensus on relative discriminatory ability or difference in calibration when comparing alternative models.^19^

Models from high-income settings such as Clinical Risk index for Babies include predictors that are not available in our data set, which is likely the best in Kenya at the time.^7 20^ Houweling *et al* 21 published models for neonatal mortality from India, Nepal and Bangladesh. Besides the Simplified Age–Weight–Sex score and the NMR-2000, these appear to be the only models applicable to LMIC settings published to date.^7 22 23^ They used population surveillance data, which differ from NETS and SENSS, which used routine hospital data.^12^ In addition, time of death encompassed 0–28 days in contrast to NETS and SENSS, where only deaths occurring in the neonatal unit (most occurring in the first week) were considered. These are potentially applicable to the Kenyan context but are prediction of neonatal deaths in the general population rather than in-hospital. The Simplified Age–Weight–Sex model was developed for neonates with gestational age of ≤33 weeks and a birth weight ≤1500 g, precluding further comparison with NETS and SENSS.^22^ Similarly, the NMR-2000 is restricted to a birth weight of ≤2000 g, age less than 6 hours and includes pulse oximetry, which may not be routinely available.^23 24^

NETS and SENSS at derivation had a good ability to distinguish between neonates who died in hospital and those who did not (c-statistics of 0.92, 95% CI 0.90 to 0.93 for NETS and 0.91, 95% CI 0.89 to 0.93 for SENSS) (figure 1). Houweling *et al* derived models based on four time points: at the start of pregnancy, start of delivery, after birth and start of delivery (including multiple delivery). Only the after-birth model demonstrated an area under the curve (AUC) greater than 0.8 (pooled average 0.83, 95% CI 0.80 to 0.89).^21^

At internal validation, the optimism adjusted c-statistic was 0.916 (NETS) and 0.901 (SENSS) compared with the original 0.918 and 0.902, respectively. Similarly, there was minimal change in the respective calibration intercept and slope (table 2). In our systematic review on neonatal treatment intensity scores, none of the 10 studies included performed internal validation.^7^ Houweling and colleagues conducted cross-validation and again only the after-birth model had an AUC greater than 0.8 (pooled average 0.83, 95% CI 0.79 to 0.86).^21^

The most useful aspect of prediction model performance is in an external population to assess generalisability (external validation).^17^ After temporal external validation, SENSS had a better calibration slope (0.90, 95% CI 0.78 to 1.01) than NETS (0.76, 95% CI 0.65 to 0.87). This means that across the range of predicted risks, the SENSS predictions were closer to the observed outcomes in the external validation population. However, there was deterioration in performance at external validation for both models. Three factors may explain this: (1) differences in definition and measurement of predictors and outcomes, (2) differences in the case mix and (3) inclusion of fewer patients in validation compared with derivation.^25^

The prescription of treatments (NETS predictors) and recognition of signs and symptoms of severe illness (SENSS predictors) are different clinical skills. It is possible a systematic difference in practices as junior clinicians changed contributed to the difference in model performance. In addition, the proportion of deaths per predictor for both models were less in the external validation data set (online supplemental tables 6 and 7). However, the magnitude of these differences varied between NETS and SENSS data sets, which might have contributed to the differences in model calibration observed at external validation. Finally, the validation data sets were smaller and spanned a shorter period (7 months vs 18 months). This might have resulted in case-mix difference due to random variation.

### Limitations

Variation in treatments may also be influenced by time, availability of resources and level of care (eg, intensive care).^7^ However, the use of standard treatment guidelines reduces this variation, and the model may be updated as practice changes.^6 7 9^ The missingness of SENSS predictors necessitated multiple imputation. It is hard to eliminate missingness in large observational data sets, and imputation provides an approach to manage this while trying to continuously improve data quality through interventions like audit and feedback.^12 26^ Temporal external validation of NETS and SENSS does not translate to generalisability in other neonatal units as this can only be assessed by externally validating the model with such external data (geographical external validation). However, there exists within Kenya the potential to conduct geographical external validation using routine data from other neonatal units.^27^ After validation, application to individual patients should be preceded by impact studies (preferably cluster randomised trials) that evaluate effectiveness and safety.^6^

### Implications

Using routine neonatal data in a low-resource setting, we found that it may be possible to predict in-hospital mortality using either treatments or signs and symptoms. Using treatments as predictors (NETS) had the advantage of availability of data in contrast to the signs and symptoms (SENSS), which required imputation. Prediction of in-hospital mortality can be used for case-mix adjustment and potentially to inform treatment decisions for individual patients (such as referral to higher-level facilities based on risk of death). Case-mix adjustment of in-hospital mortality is the most important as this is a vital component in the exploration of health system performance at scale in the delivery of care for small and sick neonates. The NETS model may be more suited for case-mix adjustment, given our experience that that it is much easier to identify treatment data than clinical signs where data can only be obtained from retrospective data.

## Data Availability

Data are available upon reasonable request. The source data are owned by the Kenyan Ministry of Health, County Governments and as the data might be used to deidentify hospitals the study authors are not permitted to share the source data directly. Users who wish to reuse the source data can make a request initially through the KEMRI-Wellcome Trust Research Programme data governance committee. This committee will supply contact information for the KEMRI Scientific and Ethical Review unit, County Governments and individual hospitals as appropriate. The KEMRI-Wellcome Trust Research Programme data governance committee can be contacted on: dgc@kemri-wellcome.org.
