# Supplementary material for: Prediction modelling of inpatient neonatal mortality in high-mortality settings
Source: Arch Dis Child. 2020 Oct 22;106(5):449–54. doi: 10.1136/archdischild-2020-319217 (PMC8070601; doi:10.1136/archdischild-2020-319217)
Supplement: Supplementary data [file archdischild-2020-319217supp001.pdf]

Supplementary Table 1: Essential treatments prescribed at admission that were included as predictors

Variable name	Description	Definition	
		Yes	No*
**Intravenous antibiotics**	Penicillin AND gentamicin are first line antibiotics prescribed together for possible Severe Bacterial Infection.	Prescribed	Not prescribed
**Intravenous fluids**	Any prescription of an infusion of intravenous fluid e.g. 10% Dextrose	Prescribed	Not prescribed
**Enteral feeding**	Expressed breast milk or formula fed through nasogastric tube or cup	Prescribed	Not prescribed
**Oxygen**	Supplemental oxygen provided by any method e.g. nasal cannula	Prescribed	Not prescribed
**Phenobarbital**	Prescription of phenobarbital	Prescribed	Not prescribed

*Reference category in the model

Supplementary Table 2: Symptoms and signs of severe illness considered as candidate predictors

Variable name	Description†	Definition	
		Yes	No*
**Difficulty feeding**	Symptom: reported history of difficulty feeding	Present	Absent
**Convulsions**	Symptom: reported episode(s) of convulsions	Present	Absent
**Indrawing**	Sign: severe (deep) lower chest indrawing	Present	Absent
**Central cyanosis**	Sign: bluish or greyish discolouration of the tongue	Present	Absent
**Floppy/inability to suck**	Sign: measure of altered consciousness in young infants (age 0-59 days)	Present	Absent

*Documented that the patient did not have the symptom or sign present

† Adapted from *Opiyo N, English M. What clinical signs best identify severe illness in young infants aged 0-59 days in developing countries? A systematic review. Archives of Disease in Childhood. 2011;96(11):1052-9.*

Supplementary Table 3: Clinical characteristics of neonates included in the model development data set

Characteristic	Random sample(n=5427)		NETS* development data set(n=4840)		Excluded from NETS (n=587)†	
	n	%	n	%	n	%
**1.Sex**						
**Male**	2937	**54**	2605	**54**	332	**57**
**Missing**	13	**0.2**	12	**0.3**	1	**0.2**
**2.Birthweight (kg)**						
**<1**	32	**0.6**	31	**0.6**	1	**0.2**
**1-<1.5**	136	**3**	115	**2**	21	**4**
**1.5-<2.5**	1180	**22**	1043	**22**	137	**23**
**2.5-4**	3841	**71**	3438	**71**	403	**69**
**>4**	229	**4**	204	**4**	2	**4**
**Missing**	9	**0.2**	9	**0.2**	0	
**3.Mode of delivery**						
**Spontaneous vaginal**	3011	**57**	2697	**57**	314	**55**
**Assisted vaginal**	3	**0.1**	1	**0.02**	2	**0.3**
**Breech**	43	**1**	40	**1**	3	**1**
**Caesarean section**	2212	**42**	1999	**42**	255	**44**
**Missing**	158	**3**	145	**3**	13	**2**
**4.Outborn‡**						
**Yes**	123	**2**	107	**2**	16	**3**
**Missing**	0		0	**-**	0	**-**
**5. Apgar score (5min)**						
**0-3**	116	**3**	112	**2**	-	**-**
**4-6**	602	**13**	593	**13**	-	**-**
**7-10**	3992	**85**	3918	**85**	-	**-**
**Missing**	717	**11**	217	**4**	500	**86**
**7.HIV exposure**						
**Exposed**	319	**6**	287	**6**	32	**6**
**Missing**	305	**6**	277	**6**	28	**5**
**8.Outcome**						
**Discharged alive**	4838	**89**	4318	**89**	520	**89**
**Dead**	508	**9**	447	**9**	61	**10**
**Referred**	62	**1**	56	**1**	6	**1**
**Missing**	19	**0.4**	19	**0.4**	0	**-**

* NETS: Neonatal Essential Treatment Score

† Excluded due to missing treatment sheets in the patient file

‡ Outborn refers to neonates admitted to the unit having been born either in another facility, at home or on the way to hospital

Supplementary Table 4: Comparison of patients included in NETS derivation and external validation

**Characteristic**	**NETS[*] derivation (n=4840)**		**NETS[*] external validation (n=1443)**	
	**n**	**%**	**n**	**%**
**1.Sex**				
**Male**	2605	**54**	850	**59**
**Missing**	12	**0.3**	2	**0.1**
**2.Birthweight (kg)**				
**<1**	31	**0.6**	10	**1**
**1-<1.5**	115	**2**	40	**3**
**1.5-<2.5**	1043	**22**	316	**22**
**2.5-4**	3438	**71**	1002	**69**
**>4**	204	**4**	74	**5**
**Missing**	9	**0.2**	1	**0.1**
**3.Mode of delivery**				
**Spontaneous vaginal**	2697	**57**	897	**63**
**Assisted vaginal**	1	**0.02**	0	**0**
**Breech**	40	**1**	19	**1**
**Caesarean section**	1999	**42**	509	**36**
**Missing**	145	**3**	18	**1**
**4.Outborn ‡**				
**Yes**	107	**2**	57	**4**
**Missing**	0	**0**	0	**0**
**5. Apgar score (5)**				
**0-3**	112	**2**	28	**2**
**4-6**	593	**13**	192	**14**
**7-10**	3918	**85**	1122	**84**
**Missing**	217	**4**	69	**5**
**7.HIV exposure**				
**Exposed**	287	**6**	74	**5**
**Missing**	277	**6**	80	**6**
**8.Outcome**				
**Discharged alive**	4318	**89**	1262	**88**
**Dead**	447	**9**	137	**10**
**Referred**	56	**1**	38	**3**
**Missing**	19	**0.4**	6	**0.4**
**9. Admission Diagnosis**				
**Birth Asphyxia**	2011	**42**	494	**34**
**Preterm/LBW**	979	**201**	257	**18**
**Neonatal sepsis**	495	**10**	172	**12**
**Respiratory D. Syndrome**	331	**7**	103	**7**
**Neonatal jaundice**	272	**6**	141	**10**
**Others**	513	**11**	332	**23**

* NETS: Neonatal Essential Treatment Score

Supplementary Table 5: Distribution of predictors and missingness in the SENSS datasets

Characteristic	Category	SENSS* Derivation N= 5427		External Validation N= 1627	
		n	%	n	%
**1.Sex**					
	Male	2937	54	961	58
	Missing	13	0.2	2	0.1
**2.Birthweight(kg)**					
	<1	32	0.5	10	0.6
	1-<1.5	136	3	45	3
	1.5-<2.5	1180	22	361	22
	2.5-4	3841	71	1125	69
	>4	229	4	85	5
	Missing	9	0.2	9	0.2
**3.Difficulty feeding**					
	Yes	225	5	134	9
	Missing	575	11	199	12
**4.Convulsions**					
	Yes	29	0.5	25	2
	Missing	571	11	199	12
**5.Central cyanosis**					
	Yes	222	5	64	5
	Missing	570	11	216	13
**6.Severe indrawing**					
	Yes	429	9	62	5
	Missing	620	11	234	14
**7.Floppy/unable to suck**					
	Yes	530	11	359	26
	Missing	876	16	222	13
**8.Outcome**					
	Dead	508	9	151	9
	Missing	19	0.4	6	0.4

*SENSS: Score for Essential Neonatal Symptoms and Signs

Supplementary Table 6: Distribution of NETS predictors by in-hospital mortality

Predictors	NETS* External validation(n=1443)						NETS* Development (n=4840)					
	Prescribed		Dead†		Missing‡		Prescribed		Dead†		Missing‡	
	n	%	n	%	n	%	n	%	n	%	n	%
**Intravenous antibiotics**	780	**54**	123	**16**	1	**0.1**	1892	**39**	383	**20**	4	**0.2**
**Intravenous fluids**	495	**34**	116	**23**	1	**0.2**	869	**18**	295	**34**	1	**0.1**
**Enteral feeds**	598	**41**	7	**1**	2	**0.3**	1381	**29**	11	**1**	8	**0.6**
**Oxygen**	105	**7**	105	**24**	0	**0**	295	**6**	120	**41**	0	**0**
**Phenobarbital**	72	**5**	21	**25**	1	**1.5**	69	**1**	42	**61**	0	**0**
**None**	196	**14**	9	**5**	3	**1.5**	834	**17**	18	**2**	8	**1**

* NETS: Neonatal Essential Treatment Score

† Denominators obtained by subtracting the number missing outcome data for each predictor

‡ Missing outcome data for each predictor

Supplementary Table 7: Distribution of SENSS predictors by in-hospital mortality

Predictor	SENSS Derivation (n=5427) †				External validation (n=1627) ‡			
	Present		Dead§		Present		Dead§	
	n	%	n	%	n	%	n	%
**1.Difficulty feeding**	264	**5**	110	**42**	146	**9**	42	**29**
**2.Convulsions**	38	**1**	17	**45**	29	**2**	7	**24**
**3.Central cyanosis**	255	**5**	141	**56**	79	**5**	35	**44**
**4.Severe indrawing**	494	**9**	266	**54**	69	**4**	40	**59**
**5.Floppy/unable to suck**	647	**12**	297	**46**	413	**25**	127	**31**

*SENSS: Score for Essential Neonatal Symptoms and Signs

† One of the 31 multiply imputed data sets

‡ One of the 23 multiply imputed data sets

§ Number and proportion dead amongst those with the predictor present

Supplementary Table 8: SENSS predictors in the original and imputed data sets

Characteristic	Category	SENSS* Derivation (N†=5427)				External validation (N†=1627)			
		Original		Imputed‡		Original		Imputed‡	
		n	%	n	%	n	%	n	%
**1.Sex**									
	Male	2937	**54**	2942	**54**	961	**58**	962	**59**
**2.Birthweight(kg)**									
	<1	32	**0.5**	32	**0.6**	10	**0.6**	10	**0.6**
	1-<1.5	136	**3**	136	**3**	45	**3**	45	**3**
	1.5-<2.5	1180	**22**	1182	**22**	361	**22**	361	**22**
	2.5-4	3841	**71**	3848	**71**	1125	**69**	1126	**69**
	>4	229	**4**	229	**4**	85	**5**	85	**5**
**3.Difficulty feeding**									
	Yes	225	**5**	264	**5**	134	**9**	146	**9**
**4.Convulsions**									
	Yes	29	**0.5**	38	**0.5**	25	**2**	29	**2**
**5.Central cyanosis**									
	Yes	222	**5**	255	**5**	64	**5**	79	**5**
**6.Severe indrawing**									
	Yes	429	**9**	494	**9**	62	**5**	69	**4**
**7.Floppy/unable to suck**									
	Yes	530	**11**	647	**11**	359	**26**	413	**25**
**8.Outcome**									
	Dead	508	**9**	509	**9**	151	**9**	151	**9**

* SENSS: Score for Essential Neonatal Symptoms and Signs
† Total number of patients in the data set
‡ Imputation models included auxiliary variables: mode of delivery, place of delivery, HIV exposure status and documentation of Apgar score. Outcome was also included. Multiple imputation using the chained equation approach was implemented using logistic regression for binary variables while categorical variables were imputed with polytomous logistic regression. There were no continuous variables.
